# Effects of Small-Sided Games *vs*. Interval Training in Aerobic Fitness and Physical Enjoyment in Young Elite Soccer Players

**DOI:** 10.1371/journal.pone.0137224

**Published:** 2015-09-02

**Authors:** Asier Los Arcos, Juan Sebastián Vázquez, Juan Martín, Javier Lerga, Felipe Sánchez, Federico Villagra, Javier J. Zulueta

**Affiliations:** 1 University School of Teaching, University of the Basque Country, UPV/EHU, Vitoria-Gasteiz, Spain; 2 University of Navarra School of Medicine, Pamplona, Spain; 3 Club Atlético Osasuna, Pamplona, Spain; 4 Respiratory Medicine Service, Clínica Universidad de Navarra, University of Navarra, Pamplona, Spain; 5 Sports Medicine Service, Clínica Universidad de Navarra, University of Navarra, Pamplona, Spain; University of Rome, ITALY

## Abstract

The purpose of this study was to compare the effects of Small-Sided Games (SSG) *vs*. Interval Training (IT) in soccer training on aerobic fitness and physical enjoyment in youth elite soccer players during the last 8 weeks of the season. Seventeen U-16 male soccer players (age = 15.5 ± 0.6 years, and 8.5 years of experience) of a Spanish First Division club academy were randomized to 2 different groups for 6 weeks: SSG group (n = 9) and IT group (n = 8). In addition to the usual technical and tactical sessions and competitive games, the SSG group performed 11 sessions with different SSGs, whereas the IT group performed the same number of sessions of IT. Players were tested before and after the 6-week training intervention with a continuous maximal multistage running field test and the counter movement jump test (CMJ). At the end of the study, players answered the physical activity enjoyment scale (PACES). During the study, heart rate (HR) and session perceived effort (sRPE) were assessed. SSGs were as effective as IT in maintaining the aerobic fitness in elite young soccer players during the last weeks of the season. Players in the SSG group declared a greater physical enjoyment than IT (*P* = 0.006; ES = 1.86 ± 1.07). Coaches could use SSG training during the last weeks of the season as an option without fear of losing aerobic fitness while promoting high physical enjoyment.

## Introduction

Depending on tactics, age, and playing position, young soccer players (12 to 18-year-old) can run a total distance of 6,000 to 9,000 m during a match [1,2]. Out of this total, between 350–550 m are ran at high-intensity (i.e., 16–19 km·h^−1^), and 200–650 m are ran with sprints (i.e. > 19 km·h^−1^) [1]. Using different definitions, others have found that 759 ± 437 m correspond to high intensity activities (i.e. > 13 km·h^−1^) [2]. Furthermore, they perform an average of 2.7 sprints per repeated-sprint sequences (i.e., minimum of 2 consecutive ≥ 1 s sprints interspersed with a maximum of 15, 30, 45 or 60 s of recovery) with an average sprint duration of less than 3 s. [3] In this context, cardiorespiratory fitness is, among other factors, key to respond to this physical demand [1,4], and it has usually been assessed in young soccer players [5,6].

To improve cardiorespiratory fitness, several training programmes, including aerobic conditioning and small-sided games (SSG), have been applied in young soccer training [7–12]. Although previous studies have shown that interval training (IT) and anaerobic speed endurance [7,8,11] as part of soccer training enhance or maintain aerobic fitness in young soccer players, physical trainers prefer SSG and conditioned games [i.e., match-play with reduced number of players [13]] because: a) they enhance work on technical and tactical parameters [14], and b) they elicit high HR intensities (i.e. > 90%HR_max_) [15,16] in the range of those reported to be functional in enhancing aerobic fitness in soccer players (i.e. 90–95% of HR_max_) [14]. Furthermore, it is thought that in comparison to physical conditioning, these types of training drills result in more enjoyment. However, studies specifically related to soccer training are needed to support this. Physical enjoyment is a critical aspect of the competitive youth sport experience [17]. There is evidence that athletes who enjoy sports the most are the ones who report being more intrinsically motivated [18,19]. Moreover, when young soccer players perceive that their psychological needs are satisfied, they report a higher degree of self-determined motivation [20].

In relation to physical conditioning, it has been suggested that in young soccer players SSG may be a good alternative to classical physical conditioning to maintain or improve aerobic fitness after pre-season and during the season. Hill-Hass et al. [9] have shown that SSG and generic training are equally effective at improving pre-season YYIRTL1 (i.e., Yo-Yo Intermittent Recovery Test Level 1) performance. Impellizeri et al. [10] observed that SSG and aerobic IT were also equally effective on aerobic fitness after pre-season and after additional 8 weeks of training. Reilly et al. [12] reported that after a 6-week program during the competitive period, the effects on aerobic capacity were similar between SSG and IT. Radziminski et al. [21] found that in young soccer players, SSG training was more effective in improving VO_2max_ than an IT protocol. However, to our knowledge, no study has compared the effects of SSG *vs* IT in young elite soccer players just during the last weeks of the season, during the last competition phase, where a tendency for a decline in aerobic fitness has been described in young [22], amateur [23], and professional elite [24–26] soccer players.

The purpose of this study was to compare the effects of SSG *vs* IT on aerobic fitness and physical enjoyment in elite youth soccer players during the last weeks of the season. We hypothesized that SSG are as effective as IT to maintain aerobic fitness and that the SSG promote higher physical enjoyment than IT.

## Materials and Methods

### Experimental Approach to the Problem

Even though several studies have compared the effects of SSG and other training methods (i.e., generic and interval training) on physical performance [9,10,12], to our knowledge, this comparison has not been carried out during the last weeks of the season in elite young soccer players. This study was done over 8 weeks, including a 6-week training period (last 6 weeks of the season) and 1+1 weeks of testing (before and after the 6-week training period). Before and immediately after the 6-week training sessions, anthropometric values, aerobic fitness [27], and counter movement jump (CMJ) [28] were assessed on all the players. In addition, heart rate (HR) and muscular and respiratory perceived effort [29–31] were also registered during training and matches. Prior to the initiation of the study players did not undergo any specific conditioning program. Physical fitness was maintained with the usual training sessions and the competitive matches.

Players were randomly assigned to the SSG or the IT groups. SSG and IT sessions were done twice a week. In addition to the SSG and IT programs, all players continued to participate in their usual training sessions and official games. Each usual training session was held 3 times pppper week and were designed as follows: Monday: Opposition-free ball drill (i.e., technical drill), tactical drills and SSGs for the players that did not compete during the weekend; Wednesday: Opposition-free ball drill and Small- and Large-sided games; Friday: Opposition-free ball drill, pre-match strategy. The exact program for each SSG and IT training sessions was implemented at the onset of each session and after a standardized warm-up.

### Participants

Seventeen U-16 male soccer players (mean ± SD: age = 15.5 ± 0.6 years, stature = 177.2 ± 5.4 cm, body mass = 68.3 ± 5.1 kg, and body fat = 10.4 ± 0.7), from a Spanish First Division club academy participated in the study. All players had a minimum of 8.5 years of experience in competitive soccer and competed for the same youth category at a national level. After informed consent was given, subjects were divided into two teams and matched according to their role (defender, midfielder and forward). Allocation to either the SSG or IT groups within each pair was performed by tossing a coin [10]. After randomization, no differences between groups were found on the performance on the baseline endurance test. Goalkeepers were excluded from the study, although they participated in the training. The Research Ethics Committee of the Navarra University Clinic (Clínica Universidad de Navarra) granted approval for the study. All players and parents were notified of the research procedures, requirements, benefits and risks before giving written informed consent. Players and coaches were not aware of the study hypothesis.

### Continuous maximal multistage running field test

In order to determine the maximal aerobic speed (MAS) [i.e., the lowest velocity that elicits VO_2_max during a graded test [32]], and to estimate the maximal oxygen uptake (VO_2_max), the Université de Montréal Tract Test (UM-TT) [27] and variations of it have been commonly used in the evaluation of soccer players [6,16,33,34]. In this study, the incremental peak running velocity was assessed using the UM-TT test [27]. The UM-TT is a continuous maximal indirect multistage running field test based on the energy cost of running. This test has been suggested as a valid and reliable way to estimate VO_2_max [27]. Briefly, the speed of the multistage is initially set at 6.00 km·h^-1^. The speed is increased subsequently by 1.20 km·h^-1^ in 2-minute intervals. Players run guided by cones placed at specific sites of the field following instructions helped by whistles. The test is stopped when the subject was at least 9 meters behind the appropriate cone at the sound signal or felt that he could not complete the stage [27]. The velocity of the last 1-min stage completed by the subjects was retained as the players’ MAS (km·h^-1^). If the last stage was not completed entirely, the MAS was calculated using the formula of Kuipers et al. [35]: MAS = Sf + (t/60·0.5), where Sf was the last completed speed in km·h^-1^ and t in the time in seconds of the uncompleted stage.

### CMJ test

Players’ CMJ heights were tested using a switching mat (Optojump, Microgate, Spain) according to the procedures proposed by Bosco et al. [28] Players performed three jumps and the best jump was recorded. Recovery time between trials was 20s [36]. Vertical jump standardization was achieved by requiring a 90 degree knee bend, keeping hands on the waist throughout the jump, avoiding undue lateral and frontal movements, and landing with extended legs. Any jump that did not meet the established criteria was excluded from calculations and was repeated.

### Training programs

Throughout the study, all subjects trained two to three days each week (one session per day, 50–75 min per session). After 7 min of collective ball drills not involving opposition, both groups trained simultaneously completing either SSG or IT for 25 minutes. Then all players trained together and with the same program (soccer drills) for the rest of the training session.

### Small-Sided Games

The SSG training program was planned and implemented by the coach. The only guidelines provided to the coach by the research team were to match the playing duration of the SSG (i.e., 3 bouts x 4 minutes with a rest between bouts of 3 minutes [37]. Scores were considered valid only if made with the first touch, and in all SSGs the relative pitch size was 85 m^2^ (Table 1). Goalkeepers were considered for the calculation of the relative pitch size, while floaters (players off the field) were excluded. All SSGs were played with coach encouragement and without the offside rule being enforced.

**Table 1:** Small-sided Games training program.

W	S	Format	Goals	Touches
**1**	*1*	(4 vs 4) + 2F_off_	2 mini-goal	no restriction / F: 2 touches
	*2*	(4+G) vs (4+G) + 2F_off_	2 official	no restriction / F: 2 touches
**2**	*1*	(4 vs 4) + 2F_off_/1F_in_	2 mini-goal	3 touches / F: 2 touches
	*2*	(4+G) vs (4+G) + 2F_off_	2 official	3 touches / F: 2 touches
**3**	*1*	(4+G) vs (4+G) + 2F_off_/1F_in_	2 official	3 touches / F: 2 touches
**4**	*1*	(4 vs 4) +1F_in_	4 mini-goal	no restriction
	*2*	(4+G) vs (4+G) + 1F_in_	2 official	no restriction
**5**	*1*	(3 vs 3) + 1F_in_	4 mini-goal	no restriction
	*2*	(4+G) vs (4+G) + 2F_off_	2 official	no restriction
**6**	*1*	(4 vs 4) + 1F_in_	4 mini-goal	no restriction
	*2*	(4+G) vs (4+G)	2 official	no restriction

W = Week; S = Session; G = Goalkeeper; F = Floater; F_off_ = Floater off field; F_in_ = Floater in the field

### Interval Training

The aerobic training intervention consisted of 3 bouts of 4 min each of running at an exercise intensity of 90–95% of HR_max_ for each player, separated by 3 minute active resting periods of jogging at 50–60% of HR_max_ [8].

### Training load quantification

Heart rate (HR) was recorded every 5 s during all training sessions using short-range telemetry (Polar Team Sport System 2, Polar Electro Oy, Kempele, Finland). No HR was recorded during matches due to the prohibition of wearing HR monitors and belts during official competitive matches. To reduce HR recording error, all players were regularly asked to check their HR monitors during each training session. Training-HR data was expressed as percentage of HR_max_ and classified into three intensity zones [9]: < 80%, 80–90%, and > 90% of HR_max_. Time spent (in minutes) in each training intensity was recorded. The HRmax reached during the first UM-TT was used as the reference to establish the HR intensity zones.

Players reported their session-perceived effort (sRPE) using the 0–10 point Borg category RPE scale [38], modified by Foster et al. [39], approximately 10 min after each training and after each official match [29,31]. Athletes were allowed to mark a plus sign (interpreted as 0.5 point) alongside the integer value [29,31]. This scale was applied during the previous four months so players were highly familiarized with the scale. Using the Foster 0–10 point scale, players were asked by the physical trainer on all occasions to rate their perceived level of effort separately for respiratory and lower extremity musculature effort [16,29–31]. These were labelled as session respiratory perceived effort (sRPEres) and muscular perceived effort (sRPEmus). Then, sRPE training load (TL), sRPE-TL, was calculated multiplying sRPE by the duration of the training or match in minutes [39]. Thus, we recorded respiratory sRPE-TL (sRPEres-TL) and muscular sRPE-TL (sRPEmus-TL). The duration of a training session was recorded for each player from the start to the end of the session, including recovery periods but excluding stretching exercises. The match duration excluded the warm-up and the in-between half-time rest.

### Physical Activity Enjoyment Scale (PACES)

After the training program, all players completed the Physical Activity Enjoyment Scale (PACES) [40]. Specifically, we used the PACES modified to the Spanish context [41]. These authors demonstrated the validity and reliability of this tool in quantifying sport enjoyment in young males [41].

### Statistical Analysis

Results are presented as means ± SD. Data normality was assessed using the Shapiro-Wilk test. Between groups differences at baseline were assessed using unpaired samples t-tests. In order to assess the difference between both groups in the time spent in the selected training zones, sRPEres-TL and sRPEmus-TL an impaired t-test was applied. The between groups comparison of post-test was performed using Analysis of Covariance (ANCOVA) including the baseline observation as a covariate. Intra-group differences from pre-test and post-test were calculated using paired t-test. Practical significance was also assessed by calculating the Cohen’s d effect size [42]. Effect sizes (ES) < 0.2, 0.2–0.6, 0.6–1.2, 1.2–2.0, 2.0–4.0 and 2.0–4.0, were considered as trivial, small, moderate, large and very large, respectively [43]. Probabilities were also calculated to establish whether the true (unknown) differences were lower, similar or higher than the smallest worthwhile difference or change (0.2 multiplied by the between-subject SD, based on Cohen's effect size principle). Quantitative chances of higher or lower differences were evaluated qualitatively as follows: < 1%, almost certainly not; 1−5%, very unlikely; 5−25%, unlikely; 25−75%, possible; 75−95%, likely; 95−99%, very likely; > 99%, almost certain. If the chance of having higher or lower values than the smallest worthwhile difference were both > 5%, the true difference was assessed as unclear. Training load data was analysed using an independent samples t-test. Statistical significance was set at *P* < 0.05. Data analysis was performed using the Statistical Package (version 18.0 for Windows, SPSS Inc., Chicago, IL, USA) and a modified statistical Excel spreadsheet [44].

## Results

Two out of the 17 subjects did not finish the study and were excluded from the final analysis; one from the IT group due to muscular injury in a match after Pre-Test and before the first day of the intervention program, and the other from the SSG group due to category promotion after two intervention sessions had been completed. Therefore, 15 subjects were included in the final analysis (Table 2) (data in S1 Dataset).

**Table 2:** Physical characteristics of the players.*

Group	n	Age	Height (cm)	Weight (kg)	Body Fat (%)
**IT**	8	15.8 ± 0.5	177 ± 5	69 ± 6	10.9 ± 0.7
**SSG**	7	15.1 ± 0.7	176 ± 6	67 ± 5	10.1 ± 0.5

IT = Interval Training; SSG = Small-sided Game.

*Values are mean ± SD

As Table 3 (data in S1 Dataset) shows, within-group practical effect for MAS from T1 to T2 was only possibly small or trivial for IT and SSG group, respectively. No significant group effect was detected in MAS (*P* > 0.05) after ANCOVA analysis. In relation to CMJ jump, there was not substantial within group practical effect for the IT group (from 42.76 ± 4.59 to 42.41 ± 4.76; *P* = 0.477; ES = -0.07 ± 0.17, likely 1/90/9), and only a possible small impairment was observed for SSG group (from 42.71 ± 2.43 to 41.96 ± 2.76; *P* = 0.100; ES = -0.25 ± 0.25, possibly 1/34/65). No significant group effect was detected in CMJ (*P* > 0.05) after ANCOVA analysis.

**Table 3:** Results, change in mean (%) and effect size (ES) of MAS (km*h^-1^) from Test 1 (T1) to Test 2 (T2).

Group	n	T1	T2	Change (%)	*p*	ES	Rating	
IT	8	16.8 ± 0.9	17.1 ± 1.0	1.7 ± 1.5	0.08	0.27 ± 0.25	Possibly	69/30/0
SSG	7	17.0 ± 0.8	16.9 ± 0.8	-0.4 ± 1.9	0.72	-0.07 ± 0.34	Possibly	9/68/23

IT = Interval Training; SSG = Small-sided Game.

After the 6 weeks of the intervention, possibly small or trivial differences were found between the IT and the SSG groups in total sRPEres-TL (8108 ± 1472 vs 7891 ± 1306 AU, *P* = 0.77; ES = 0.32 ± 0.47, possibly 4/28/69) and sRPEmus-TL (8510 ± 1131 vs 8447 ± 2218 AU, *P* = 0.94; ES = 0.16 ± 0.56, possibly 13/42/45), respectively. In relation to time spent at different intensities during training sessions, players in the SSG group accumulated a likely greater training time (12.7 ± 6.4 vs 7.2 ± 3.8%; *P* = 0.07; ES = 1.31 ± 1.30, likely 92/4/3) at high intensity (> 90% of HR_max_) than in the IT group, but likely moderate lower training time (19.4 ± 5.3 vs 22.6 ± 2.8%; *P* = 0.16; ES = -1.03 ± 1.33, likely 6/8/86) at medium intensity (80–90% of HR_max_), and small lower practice time (68.0 ± 10.6 vs 70.3 ± 5.3%; *P* = 0.60; ES = -0.38 ± 1.38, possibly 23/18/59) at low intensity (< 80% of HR_max_).

Very likely large practical differences (*P* = 0.006, ES = 1.86 ± 1.07, very likely 99/1/0) were found between SSG (28.43 ± 9.11) and IT (15.63 ± 6.12) groups in the Physical Activity Enjoyment Scale (PACES) results (Table 4) (data in S1 Dataset).

**Table 4:** Results of Physical Activity Enjoyment Scale (PACES) (arbitrary units).

	ITG	SSGG	*p*	ES	Rating	
**PACES Scores**	15.63 ± 6.1	28.43 ± 9.1	0.006	1.86 ± 1.07	Very Likely	99/1/0

ITG = Interval Training Group; SSGG = Small-sided Game group; ES = Effect Size.

## Discussion

The purpose of this study was to compare the effect of SSG *vs* IT on aerobic fitness and physical enjoyment in elite youth soccer players. The main findings of our study were: a) SSG and IT were equally effective in maintaining aerobic fitness in elite young soccer players during the last weeks of the season, and b) SSG promoted considerable higher physical enjoyment than IT.

After the 6-week training period during which SSG or IT training were added to the habitual soccer practice, no significant group effect was detected in MAS, and only possibly small practical and positive effect was found for the IT group in this physical fitness parameter (Table 3). Therefore, in elite youth soccer players both training programs were equally effective at maintaining cardiorespiratory fitness during the last weeks of the season. These results are in concordance with previous studies performed during pre-season and at the start of the in-season period [9,10,12,21], but to our knowledge this is the first time the comparison between SSG and IT has been during the last weeks of the season, when aerobic fitness in soccer players, at most, is maintained [22–26,45]. Thus, technical and tactical parameters could be worked in young soccer players with SSG during the last weeks of the season without impairment in cardiorespiratory fitness. Furthermore, in relation to neuromuscular performance, no significant group effect was detected in CMJ, and only possibly small practical impairment was found for SSG group. Thus, it is not likely that SSGs have a negative effect on neuromuscular performance during the last weeks of the season. These results suggest that both cardiorespiratory fitness and jump capacity can be maintained during the latter part of the season by including soccer drills to the habitual training routine.

Taking into account that players’ practice was not limited to the designed intervention programs, we quantified TL during total practice time in both groups since this can affect changes in aerobic fitness [46–49]. While non-significant and only possibly small differences were found between both groups regarding total respiratory and muscular perceived TL, according to HR data, players in the SSG group accumulated likely moderate greater training time (12.7 ± 6.9 vs 7.2 ± 3.8%; ES = 1.31 ± 1.30) at high intensity than in the IT group. Previous studies with soccer players found linear and positive dose-response relationships between training accumulated at high intensity and changes in different aerobic fitness parameters [47,48]. After 6 to 8 weeks of pre-season training in elite soccer players, these reports found large to very large associations between the training time spent at high intensity (i.e., > HR at 4 mmol.L^-1^ lactate threshold) and the changes in speed at blood lactate concentrations of 2 and 4 mmol.L^-1^ [47,48], in maximal oxygen uptake [48], and in Yo-Yo IR1 [48]. However, in our study players that accumulated more training time at high intensity (i. e., SSG group) did not improve their cardiorespiratory fitness. This suggests that in young soccer players training time accumulated at high intensity may not be relevant during the last weeks of the season.

In addition to the maintenance of aerobic fitness, to our knowledge this is the first study that shows that young elite soccer players are more motivated during SSG than performing physical training (Table 4). This may be due to the fact that the activity in SSG resembles real football, which may motivate players to strive to achieve and experience greater feelings of competence than with IT. This is relevant in training because the positive emotion of enjoyment has been shown to be an important ingredient of motivation in youth and elite sports [50]. In addition, athletes who enjoy sports the most are the ones who report being more intrinsically motivated [18,19] Specifically in young soccer players, psychological need satisfaction provides the essential ingredient for self-determined motivation [20]. Therefore, in adolescents the SSG training may provide an activity stimulus that has potential psychological benefits that can help football coaches at any time during the season.

This study has some strengths and limitations. The main limitation is the small number of players in each group. Most published studies have the same limitation due to the difficulty in recruiting a homogeneous large group of players training under the same conditions. Even elite Football Clubs have one team per category, precluding the recruitment of more than 20 players at most. However, in our opinion the fact that the study was performed with players from a First Division Soccer academy following rigorous training programs with close monitoring and observation of the sessions is perhaps its major strength.

In conclusion, during the last weeks of the season, SSG training in addition to the training of the technical and tactical aspects, maintains cardiorespiratory fitness and promotes a high level of enjoyment in youth elite soccer players.

## Practical Applications

The technical staff can use SSGs to improve the soccer competence of the players during the last weeks of the season in young elite soccer players, without fear of losing aerobic fitness and providing a high physical enjoyment.

## Supporting Information

S1 Dataset (XLSX)
